# Stability Study of an Electrothermally-Actuated MEMS Mirror with Al/SiO_2_ Bimorphs

**DOI:** 10.3390/mi10100693

**Published:** 2019-10-12

**Authors:** Peng Wang, YaBing Liu, Donglin Wang, Huan Liu, Weiguo Liu, HuiKai Xie

**Affiliations:** 1 School of Optoelectronic Engineering, Xi’an Technological University, Xi’an 710021, China; hurrywp@gmail.com (P.W.); kikey7758@163.com (D.W.); liuhuan360@163.com (H.L.); 2 Wuxi WiO Technologies Co., Ltd., Wuxi 214000, China; lyb_1992@126.com; 3 Department of Electrical and Computer Engineering, University of Florida, Gainesville, FL 32611, USA; hkxie@ece.ufl.edu

**Keywords:** electrothermal actuation, electrothermal MEMS mirror, electrothermal bimorph, stability, temporal drift

## Abstract

Electrothermal actuation is one of the main actuation mechanisms and has been employed to make scanning microelectromechanical systems (MEMS) mirrors with large scan range, high fill factor, and low driving voltage, but there exist long-term drifting issues in electrothermal bimorph actuators whose causes are not well understood. In this paper, the stability of an $\mathrm{Al}/{\mathrm{SiO}}_{2}$ bimorph electrothermal MEMS mirror operated in both static and dynamic scan mode has been studied. Particularly, the angular drifts of the MEMS mirror plate were measured over 90 h at different temperatures in the range of 50–150 °C. The experiments show that the temporal drift of the mirror plate orientation largely depends on the temperature of the electrothermal bimorph actuators. Interestingly, it is found that the angular drift changes from falling to rising as the temperature increases. An optimal operating temperature between 75 °C to 100 °C for the MEMS mirror is identified. At this temperature, the MEMS mirror exhibited stable scanning with an angular drift of less than 0.0001°/h.

## 1. Introduction

Electrothermal actuation based on thermal bimorphs has been widely applied on scanning microelectromechanical systems (MEMS) mirrors due to its advantages of large displacement, high fill factor, and economical fabrication process [1]. Thermal bimorphs refer to cantilever beams composed of two structural layers with different thermal expansion coefficients (TECs). The motion is achieved by heating the thermal bimorphs coupled with the TEC difference of the two materials in the bimorphs. Aluminum and $\mathrm{Al}/{\mathrm{SiO}}_{2}$ are the most frequently-used bimorph materials as they are common materials in semiconductor fabrication and their TEC difference is large. $\mathrm{Al}/{\mathrm{SiO}}_{2}$ electrothermal bimorph-based MEMS mirrors have been extensively studied [2], and have also been used in many applications, such as endoscopic optical imaging [3] and microspectrometers [1]. However, feedback control is often needed for electrothermal MEMS mirrors in high-precision applications because of the relatively low repeatability and stability of $\mathrm{Al}/{\mathrm{SiO}}_{2}$ bimorph actuators [4]. For example, in the MEMS Fourier transform (FT) spectrometer application, the electrothermal MEMS mirror usually oscillates around a center position providing the zero optical path difference for the FT spectrometer [1]. The relatively low repeatability of the electrothermal MEMS mirror may introduce phase shift, which reduces spectral accuracy [1,5]. In optical switching, as high precision of the mirror tilt angle is required, the low stability of the MEMS mirror may increase the insertion loss dramatically [6]. Furthermore, because the influence of the factors of instability can be accumulated, the eventual mirror drift can be significantly large in the long term. To compensate the instability of the electrothermal MEMS mirrors, closed-loop control with additional optical position sensing mechanisms is generally required, which increases not only the complexity but also the size and cost. Therefore, improving stability of the electrothermal MEMS mirrors is critical for them to be used in high-precision applications.

MEMS stability depends on the combination of multiple failure mechanisms, such as creep, stress relaxation, and fatigue [7,8,9,10]. Such mechanisms are affected by material properties, fabrication process, environment, temperature, stress, working modes of MEMS devices, etc. [11,12]. Compared to dielectrics, metals are more prone to suffering from low reliability issues, especially aluminum because of its weak atomic bonds. Creep is one main failure mechanism of Al devices, where the strain continually increases under the influence of stresses until fracture. Although creep normally is noticeable when the temperature is higher than 35% of the material melt point, the creep of Al thin films is not negligible even at low temperature especially when Al is under high-level stress [13,14,15]. Stress relaxation is another issue, where the stress in a structure tends to decrease under a constant strain [8,13]. Stress relaxation is a result of plastic deformation that is a function of temperature and stress, so it is usually considered together with creep. Stress relaxation may be not a fatal problem for certain single-layer MEMS devices, but it affects the stability of bimorph actuators significantly as a bimorph actuator’s displacement depends on the stress difference between two layers. Fatigue causes permanent plastic deformation or even cracks under an alternate stress or strain. It can be expected that stress, temperature, and cycling times are the effect factors of fatigue [9,16,17]. Low stress and temperature are difficult to be achieved in electrothermal-actuated MEMS mirror as the bimorph beam bends with a significant change in stress at an elevated temperature. The reliability of an $\mathrm{Au}/{\mathrm{SiO}}_{2}$ bimorph temperature sensor was reported, and the result shows an acceptable reliability for nonprecision applications [18], but the maximum stress in this case is much less than those in electrothermal $\mathrm{Al}/{\mathrm{SiO}}_{2}$ bimorph MEMS mirrors.

In this paper, the stability of an $\mathrm{Al}/{\mathrm{SiO}}_{2}$ electrothermal MEMS mirror has been studied. The temporal angular orientation stabilities of the mirror plate under various environmental temperatures and a range of direct current (DC) and alternating current (AC) driving signals have been measured. Based on the results, several suggestions for improving stability of $\mathrm{Al}/{\mathrm{SiO}}_{2}$ electrothermal MEMS mirrors are given. In the following, the design and basic characteristics of the MEMS mirror are introduced first. Then, the experiment design and experimental results are presented. After that, the results are discussed and analyzed through simulation.

## 2. Electrothermal Bimorph MEMS Mirror

**Figure 1:**
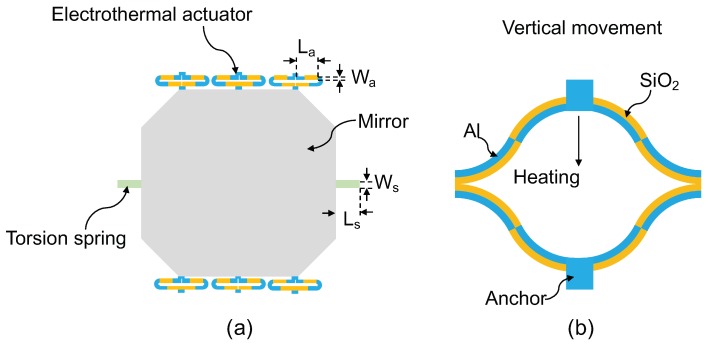
(**a**) Schematic of the electrothermal microelectromechanical systems (MEMS) mirror. (**b**) Side view of the bilaterally symmetrical folded double S-shaped bimorph (FDSB) electrothermal actuator.

To study the stability of electrothermal bimorph MEMS mirrors, one MEMS mirror with $\mathrm{Al}/{\mathrm{SiO}}_{2}$ bimorph electrothermal actuators has been used. The schematic of the MEMS mirror is shown in Figure 1a. It consists of a 2.5mm×2.5mm Al-coated silicon mirror plate, two $\mathrm{Al}/{\mathrm{SiO}}_{2}$ torsional springs, and six $\mathrm{Al}/{\mathrm{SiO}}_{2}$ electrothermal actuators. One end of each spring ($L_{s}=100\;\mu$m, $W_{s}=20\;\mu$m) is anchored on the silicon substrate; while the other ends of the springs are connected to two opposite sides (right and left) of the mirror (20-μm thick), respectively. The actuators are connected to the top and bottom sides of the mirror plate with three actuators on each side. The actuators on the top and bottom sides have reversed-order bimorph structures, so one side bends up while the other side bends down, resulting in a rotation of the mirror plate around the torsional springs as the temperature changes. The length ($L_{a}$) and width ($W_{a}$) of actuator are 330μm and 25μm, respectively. Figure 1b shows the side view of the $\mathrm{Al}/{\mathrm{SiO}}_{2}$ electrothermal actuators. The actuator is bilaterally symmetrical and each side has a folded double S-shaped bimorph (FDSB) structure consisting of four alternating bimorph sections. Each section consists of a 1.1-μm thick Al layer and 1.2-μm thick plasma enhanced chemical vapor deposition (PECVD) ${\mathrm{SiO}}_{2}$ layer. A 200-nm thick Ti resister is sandwiched in the middle of the bimorph as a heater. The Ti heater can be used to heat the actuator by applying a voltage on it.

**Figure 2:**
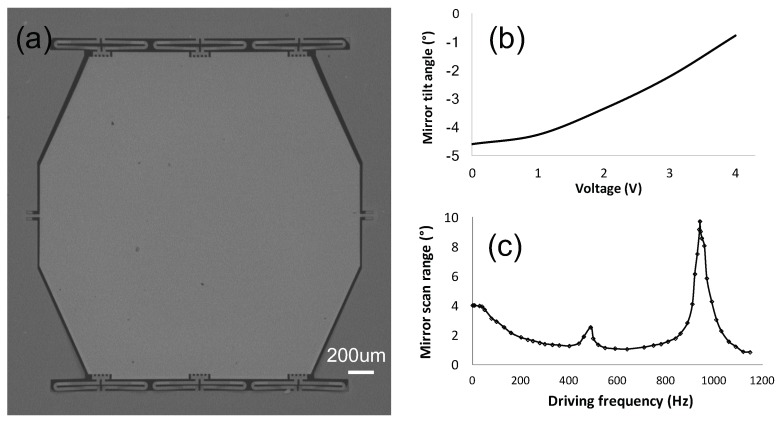
(**a**) Scanning electron microscope (SEM) image of the MEMS mirror. (**b**) Quasi-static angle of the MEMS mirror vs. voltage response. (**c**) Frequency response of the MEMS mirror.

**Figure 3:**
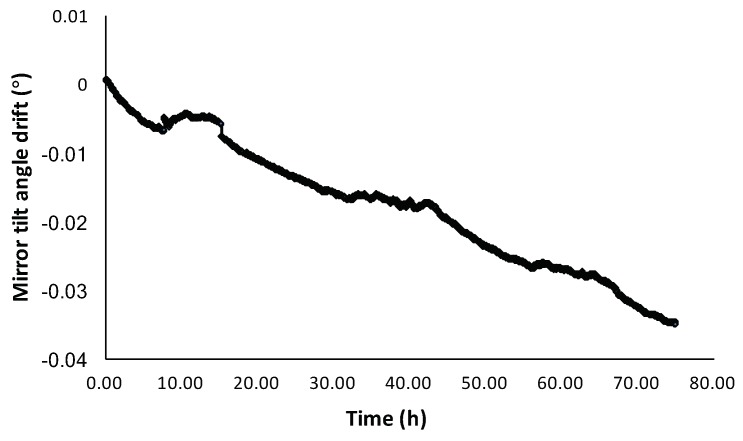
The mirror tilt angle drift as a function of time at room temperature.

Figure 2a shows the SEM image of a fabricated MEMS mirror. The fabrication process is similar to that reported in [19]. According to the process monitoring during the device fabrication, the Al and PECVD ${\mathrm{SiO}}_{2}$ layers in the bimorphs have about 160 Mpa tensile residual stress and about 350 Mpa compressive residual stress, respectively. Hence, the mirror is initially tilted with an angle, and the mirror plate tends to be flat as the temperature rises. The mirror tilt angle as a function of applied voltages on the Ti heater is shown in Figure 2b. It can be seen that the mirror has an initial tilt angle of −4.5° and the angle increases as the voltage rises. In the following, the direction in which the mirror angle changes from negative to $0^{{}^{\circ}}$ is defined as increasing. Figure 2c shows the frequency response of the mirror with a 4 V sinusoidal driving signal. It can be seen that the mirror has a resonant frequency of 960 Hz, and its linear scan range decreases rapidly after 50 Hz as the response time of the thermal bimorph actuators is about 10 ms. Hence, this electrothermal MEMS mirrors is typically used at low scanning frequency. The main problem of the electrothermal MEMS mirror is the angular drift in long term. Figure 3 shows the mirror tilt angle at room temperature for 70 h. It can be seen that the angle continuously decreased in 70 h with a total angular drift of 0.035°. The fluctuations in the graph were the result of environmental disturbance. The aim of this work is to study this angular drift and eventually eliminate it.

## 3. Stability Experiment

The stability issues of Al MEMS devices are usually due to creep, stress relaxation, and fatigue, and all of these failure mechanisms are temperature and stress dependent. In electrothermally-actuated MEMS mirrors, temperature determines the deformation of the bimorph actuators, which changes the stress distribution in the bimorphs. Hence, a set of experiments was designed to investigate the temperature stability of the electrothermally-actuated MEMS mirror. It can be expected that an optimal working temperature exists where the angular drift is minimized.

**Figure 4:**
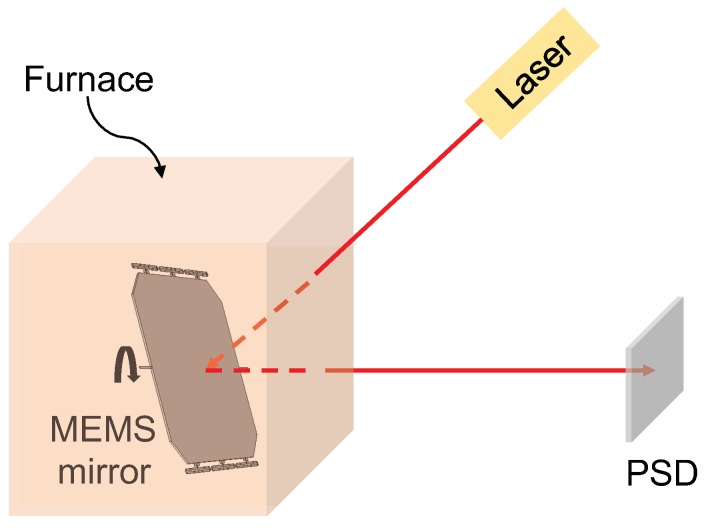
Schematic of the electrothermal MEMS mirror angular stability test setup.

To measure the angular stability of the electrothermal MEMS mirror at various temperatures, a test setup was built, as shown in Figure 4. The MEMS mirror was placed in a furnace providing various ambient temperatures. A laser (650 nm) was projected to the MEMS mirror directly without any optics, and the laser reflection was detected by a position sensitive detector (PSD). The tilt angle of the MEMS mirror can be calculated based on the reflected beam position on the PSD. Each actuator can be heated by applying a voltage on its Ti resistor.

Experiments were designed to study the static angular drifting and dynamic scan stability of the electrothermal MEMS mirror. The static drifting experiment was to study the electrothermal MEMS mirror’s angular stability at a constant temperature. In this study, the MEMS mirror chips were divided into two groups. Group I was heated by a furnace under various temperatures; while Group II was heated by the Ti resistor heaters directly.

For Group I, the MEMS mirrors were annealed at 150 °C in the furnace for a 140 h burn-in and were then cooled down to room temperature. The Al layers of the bimorph beams contain a lot of defects and nonuniform distribution of stress from microfabrication processes. Hence, the bimorphs may have different initial conditions and poor repeatability without a burn-in step. The annealing is used as the burn-in to minimize the initial variations. After that, the burnt-in MEMS mirrors were reheated in the furnace at various temperatures for angular drift measurement. The furnace temperature was increased from 50 °C to 150 °C with a step of 25 °C, and each step was held for 90 h. The laser beam position was monitored and recorded once the furnace reached each of the set temperatures.

For Group II, the same experiment was performed except that the bimorph actuators were annealed and heated by the Ti heater embedded in the bimorph instead of the furnace. The bimorph temperature is controlled by the voltage applied to the Ti resistor. The relation between the bimorph temperature and the driving voltage was extracted from the resistance measurement data and the temperature coefficient of resistance of the Ti resister. The driving voltages of 1.19 V, 1.65 V, 2.08 V, 2.50 V, and 2.85 V were used for 50 °C, 75 °C, 100 °C, 125 °C, and 150 °C, respectively. The bimorph actuator temperature was increased from 50 °C to 150 °C with a step of 25 °C, just the same as in the previous experiment. The angular drift for room temperature was measured when the actuator temperature returned back to room temperature from 150 °C after power off. At each temperature, the laser reflection from the mirror was detected by the PSD and the mirror tilt angle was recorded for 90 h.

The dynamic scan experiment was to study the reliability of the MEMS mirror oscillating under an AC driving signal. The dynamic scan reliability was examined at three offset temperatures of 62.5 °C, 100 °C, and 137.5 °C with a ±37.5 °C range. Since the scan range decreases rapidly with the frequency, as shown in Figure 2c, a 5 Hz AC voltage with various amplitudes and DC offsets was applied to the Ti resisters to actuate the mirror. Three different driving signals of 0–2.08 V, 1.43–2.67 V, and 2.08–3.24 V were used, corresponding to 25–100 °C, 62.5–137.5 °C, and 100–175 °C for the actuators, respectively. Similarly, the laser beam reflected from the mirror was picked up by the PSD and its position was recorded for more than 70 h.

## 4. Results

**Figure 5:**
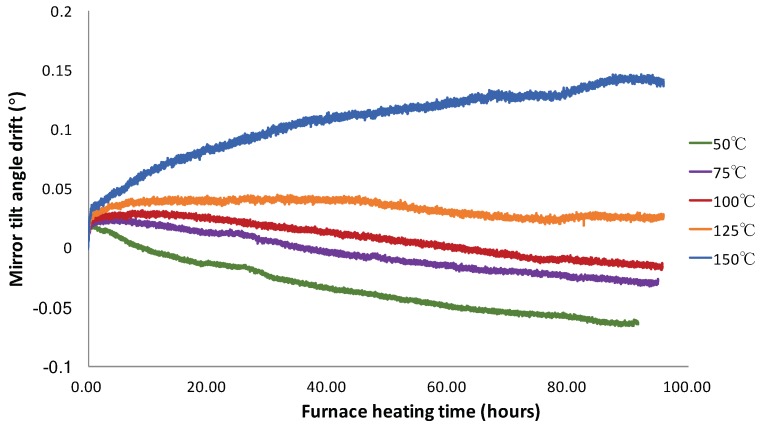
Mirror tilt angle drift as a function of time at different furnace temperatures.

In the static angular drifting study, the drift of the tilt angle of the mirror plate as a function of time at various furnace temperatures is plotted in Figure 5. As shown in the inset in Figure 5, the tilt angle increases rapidly by about 0.03° in the first hour for all temperatures. After repeating several measurements, it was found that this rapid change was because the furnace temperature was not completely settled down in the first hour. It should be noticed that 0.1° of the mirror tilt angle corresponded to a 2.2 μm displacement of the bimorph actuators. Once the chip temperature became stable, it can be seen that the angular drifting trend depended on the set ambient temperature. It can be seen that the tilt angle has a standard deviation of about 0.0005° because the furnace temperature fluctuated in a small range around the set value. It is interesting to note from Figure 5 that the drifting trend of the mirror tilt angle changed from falling to rising as the set ambient temperature was increased from 50 °C to 150 °C. In the first 10 h, the mirror tilt angle showed a slow positive drift for all temperatures above 75 °C, then the tilt angle changed from its initial rising trend to eventually a drift trend, with a relatively constant rate. At 50 °C, the tilt angle started decreasing with a constant drift rate right after the first hour. The relatively constant drift rates were −0.0009°/h, −0.0006°/h, −0.00055°/h, −0.00015°/h, and 0.001°/h for 50 °C, 75 °C, 100 °C, 125 °C, and 150 °C, respectively. The result shows that the mirror tilt angle was relatively more stable at the ambient temperature of about 125 °C.

**Figure 6:**
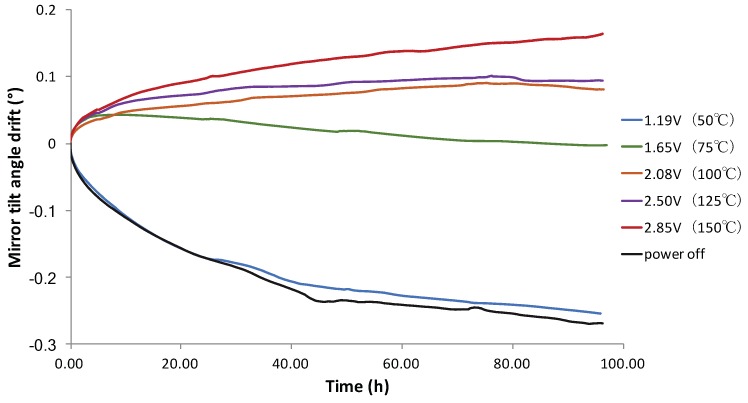
Mirror tilt angle drift as a function of time with various driving voltages.

For the MEMS mirrors in Group II, similar experiments with the MEMS mirrors under various temperatures were performed. The main difference was the different temperatures of the MEMS actuators were generated by the Ti resisters embedded in the bimorphs. The mirror tilt angle drift as a function of time with the Ti resister heating is shown in Figure 6. The Ti resister heating shows a similar angular drift trend to that in the case of the furnace heating. The tilt angle changed from falling to rising as the voltage (temperature) was increased. The tilt angle drifted fast in the first 10 h. After that, the angular drift rates were relatively constant. The angular drift rates were −0.00075°/h, −0.00078°/h, 0.00055°/h, 0.00062°/h, 0.00055°/h, and 0.00095°/h for room temperature, 50 °C, 75 °C, 100 °C, 125 °C, and 150 °C, respectively. It can be seen that the tilt angle was more stable between 75 °C and 125 °C than other temperatures.

**Figure 7:**
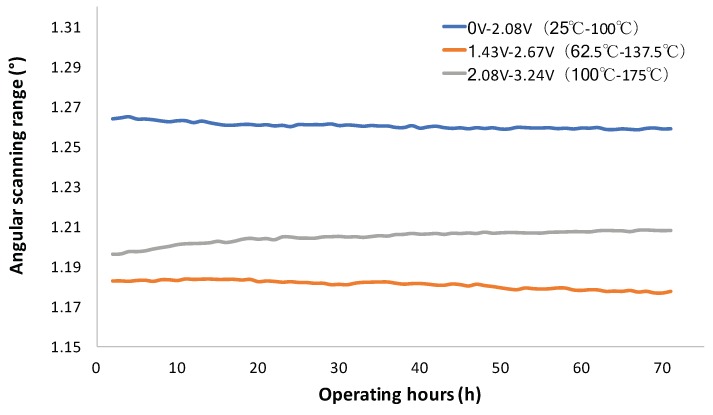
Mechanical scanning angle range of MEMS mirror driven by alternating current (AC) signals as a function of operating time.

For dynamic scan stability study, the angular ranges of the mirror scan at three different AC driving signals are shown in Figure 7. It can be seen that the scanning ranges were all quite stable in long term. However, a small decrease from 1.264° to 1.26° and a small increase from 1.195° to 1.204° were observed in the first 20 h for the signals of 0–2.08 V and 2.08–3.24 V, respectively. The angular scanning range variations were less than ±0.001°/h for all driving signals.

**Figure 8:**
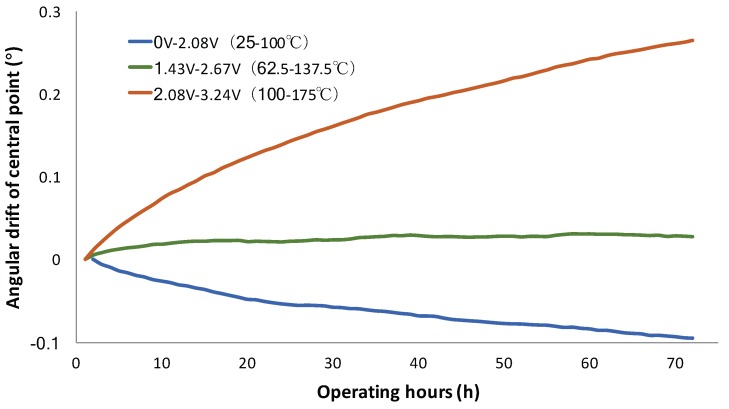
The angular drift of MEMS mirror scanning driven by AC signals as a function of operating time.

According to the data shown in Figure 7, the angular scan ranges were changing over time. Another question would be if the central points of the scan ranges changed or not. Figure 8 plots the angular positions of the central points over time when the mirror scanned under various voltage signals. The result indicates that the trend of the central point drifting under dynamic scanning is the same as that of the static angular drifting. The corresponding angle of the central point with the mirror scanning decreased when the mirror operated in the low temperature range (25–100 °C), while the angle increased in the high temperature range (100–175 °C). The angular deflection was more stable when the mirror was scanned between 62.5–137.5 °C. The angular drift rates of the central points were −0.00083°/h, 0.0001°/h, and 0.0022°/h for 25–100 °C, 62.5–137.5 °C, and 100–175 °C, respectively.

## 5. Discussion

The results have verified the hypothesis that the angular stability of $\mathrm{Al}/{\mathrm{SiO}}_{2}$ electrothermal MEMS mirrors depends on temperature. Unlike normal creep phenomena, where creep is negligible at low temperature, large angular drift was observed in the $\mathrm{Al}/{\mathrm{SiO}}_{2}$ electrothermal MEMS mirror at room temperature. In order to understand this phenomenon, COMSOL Multiphysics *©* [20], a multiphyics simulation software, was employed to look into the stresses in the bimorph structures.

**Figure 9:**
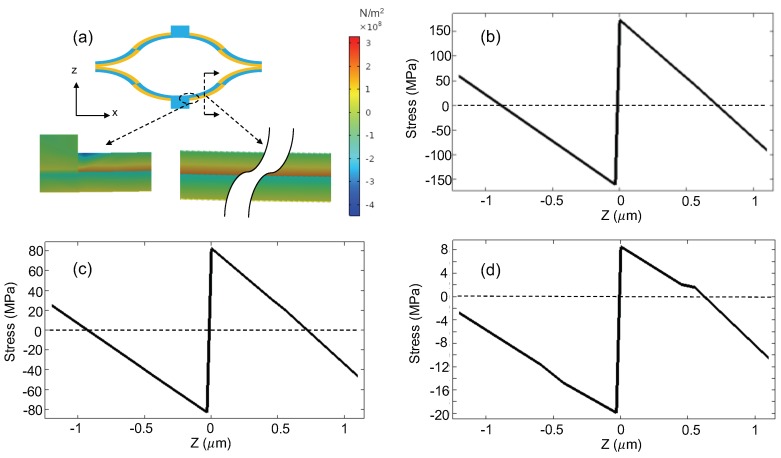
(**a**) Stress distribution on bimorph cross section at −4.5°. (**b**) Stress as a function of z-position at −4.5°. (**c**) Stress as a function of z-position at −2.5°. (**d**) Stress as a function of z-position at −0.5°.

Figure 9a shows the simulated cross-sectional stress distribution of a bimorph at 25 °C, where no voltages are applied to the actuators and the cross section is located at the middle of the first bimorph section next to the mirror connection, as indicated by the dotted line in the inset in Figure 9a. A stress concentration point with a compressive stress larger than 400 MPa is observed at the corner of the anchor (Figure 9a). However, the anchor stress does not affect the MEMS stability much, so the main focus in this paper is the stress change of the bimorph. The total thickness of the bimorph is 2.3μm. The region of −1.2μm to 0μm in the z-direction represents the ${\mathrm{SiO}}_{2}$ layer; while the region of 0μm to 1.1μm corresponds to the Al layer. As indicated by the color bar in Figure 9a, the stress changes from negative to positive multiple times. To be more quantitative, Figure 9b–d plot the stresses on the cross section as a function of the z-position at the mirror tilt angles of −4.5° (corresponding to 0 V), −2.5°, and −0.5°, respectively. It can be seen that the Al stress changes from compression to tension as the z-position is close to the $\mathrm{Al}/{\mathrm{SiO}}_{2}$ interface. At a constant temperature, it can be expected that the Al layer away from the $\mathrm{Al}/{\mathrm{SiO}}_{2}$ interface is compressed while the Al layer close to the ${\mathrm{SiO}}_{2}$ is stretched with the stress distribution shown in Figure 9b due to creep. Hence, the bimorph tends to bend more, which leads to a larger negative tilt angle. On the other hand, the stresses in all of the bimorph actuators slowly decrease due to stress relaxation, causing the mirror plate to move slightly towards its flat position (i.e., $0^{{}^{\circ}}$). Thus, stress relaxation leads to an increase in the angle, while the creep in the bimorph is significant at room temperature due to large stress in the Al layer. Figure 4b–d indicate that the stress decreases dramatically with an increase in the tilt angle corresponding to a temperature rise. Compared with the Al yield strength of 140 MPa, the maximum tensile stresses are 160 MPa, 80 MPa, and 8 MPa at the $\mathrm{Al}/{\mathrm{SiO}}_{2}$ for the mirror tilt angles of −4.5°, −2.5°, and −0.5°, respectively. The −4.5° tilt corresponds to the case with the MEMS mirror at room temperature and without actuation. Thus, it is preferred to store the MEMS mirror at an elevated temperature. Although high temperature generally exacerbates creep, the creep of the Al may actually become weaker when the temperature increases in the bimorphs, which is attributed to the significant decrease in stress at an elevated temperature and the overall relatively low operation temperature. Hence, it can be seen that the angular drift rate decreases as the temperature rises from room temperature to 125 °C, and finally a stable mirror tilt angle is observed at around 125 °C as all failure mechanisms reach an equilibrium. As the temperature continues to rise, the stress relaxation dominates the angular drift as the creep is weak under low stress. As a result, a positive angular drift is observed at 150 °C, which has a similar angular drift rate at room temperature but in the opposite direction. Therefore, the temperature-dependent stability for the bimorphs is not due to the direct effect of temperature on creep, but because temperature changes the stress in the Al layer.

The mirror heated by Ti heater shows a similar phenomenon as changing ambient temperature. However, compared with the furnace heating, the temperature distribution is less even on the actuators for Ti resister heating, as the current in the resister may change with its structure. Hence, the creep rate of bimorphs may be different based on the temperature distribution. It can be seen that the mirror tilt angle drifts faster in the first 10 h. On the one hand, as the resistivity of the Ti changes with the temperature, it may take a longer time to achieve thermal equilibrium. On the other hand, the bimorph may be experiencing primary stage of creep in the first 10 h, which cause a higher drift rate. The result shows the optimal working temperature exists between 75 °C and 100 °C, which is lower than the optimal ambient temperature of 125 °C. That may be caused by local high temperature on the bimorphs. Therefore, the electrothermal-actuated mirror can be used under the optimal temperature to minimize angular drift. Furthermore, it can be suggested that the mirror is better to be stored at around 125 °C instead of room temperature for a stable initial angle.

MEMS mirrors are usually employed as dynamic scanning devices. Under dynamic operation, the actuators experience a varying temperature, so the constant optimal temperature is no longer suitable for the mirror. However, it is interesting to note that the static motion experiments show the tendency of the angular drift at low temperature is opposite to that at high temperature, so it is possible to cancel the angle drift in dynamic motion with an AC driving signal. The dynamic experiment shows a stable mirror oscillation around 100 °C in both the scanning range and central angle for more than $10^{6}$ cycles. Comparing both the static and dynamic experiments, it can be seen that the angular drift rates in both cases are similar at the temperature swings for 25–100 °C and 62.5–137.5 °C, respectively, but the angular drift in the dynamic case is much faster than that in the static case at the high temperature swing (100–175 °C). This is believed to be because the bimorph actuator fatigue is exacerbated at high temperature, so high operating temperature should be avoided in dynamic scanning. Therefore, there exists an optimal temperature range for dynamic scanning, which provides a good angular stability. In this study, only the scan range stability under dynamic scanning is investigated. In the future, the quasi-static pointing stability will be studied for optical switching applications.

According to the above experiment result, the stability mainly depends on the stress of the Al instead of temperature. The increase in temperature reduces the stress of the Al layer, which results in a weaker creep. Therefore, the stabilizing temperature can be reduced if the residual stress of the Al layer is decreased. This can be implanted through tuning the Al deposition process.

## 6. Conclusions

In this paper, the angular temperature stability of an electrothermally-actuated $\mathrm{Al}/{\mathrm{SiO}}_{2}$ bimorph MEMS mirror has been studied. The experiments show that the angular stability depends on the actuator operation temperature, and the analysis through simulation reveals that the angular drift is a combined effect of creep, thermal relaxation, stress, and fatigue. The angular drift of the tilt angle changes from falling to rising as the temperature increases, resulting in an optimal operation temperature at which good angular stability can be achieved. For the MEMS mirror tested, the optimal temperature at the static mode is around 100 °C, while high stability is also achieved at the dynamic scanning mode by driving the bimorph actuators with an offset temperature of 100 °C. Steady oscillation of the MEMS mirror with a scanning range of 1.18° and an angular drift rate of 0.0001°/h is demonstrated by driving the mirror between 62.5–137.5 °C. This stable operation can be used in applications like FTS that require high-precision scanning.
